# Population Dynamics and Air Pollution: The Impact of Demographics on Health Impact Assessment of Air Pollution

**DOI:** 10.1155/2013/760259

**Published:** 2013-05-21

**Authors:** Esben Meulengracht Flachs, Jan Sørensen, Jakob Bønløkke, Henrik Brønnum-Hansen

**Affiliations:** ^1^National Institute of Public Health, University of Southern Denmark, Øster Farimagsgade 5, 1353 Copenhagen, Denmark; ^2^CAST, University of Southern Denmark, J.B. Winsløws Vej 9 B, 5000 Odense C, Denmark; ^3^Institue of Public Health, Aarhus University, Bartholins Allé 2, Bygning 1260, Lokale 331, 8000 Aarhus C, Denmark; ^4^Institute of Public Health, University of Copenhagen, Øster Farimagsgade 5, P.O. Box 2099, 1014 Copenhagen, Denmark

## Abstract

*Objective*. To explore how three different assumptions on demographics affect the health impact of Danish emitted air pollution in Denmark from 2005 to 2030, with health impact modeled from 2005 to 2050. *Methods*. Modeled air pollution from Danish sources was used as exposure in a newly developed health impact assessment model, which models four major diseases and mortality causes in addition to all-cause mortality. The modeling was at the municipal level, which divides the approximately 5.5 M residents in Denmark into 99 municipalities. Three sets of demographic assumptions were used: (1) a static year 2005 population, (2) morbidity and mortality fixed at the year 2005 level, or (3) an expected development. *Results*. The health impact of air pollution was estimated at 672,000, 290,000, and 280,000 lost life years depending on demographic assumptions and the corresponding social costs at 430.4 M€, 317.5 M€, and 261.6 M€ through the modeled years 2005–2050. *Conclusion*. The modeled health impact of air pollution differed widely with the demographic assumptions, and thus demographics and assumptions on demographics played a key role in making health impact assessments on air pollution.

## 1. Introduction

 Air pollution is known to have detrimental effects on health as demonstrated in numerous studies [1, 2]. A number of Health Impact Assessments (HIA) have been made to quantify the impact from various components of air pollution and in a number of different geographical settings [3–6]. Most focused on the effects of particulate matter (PM) on overall mortality [3, 5, 7], although some have included effects of a range of pollutants and/or diseases and causes of death [4, 6, 8, 9]. The Apheis project [4] assessed impact of PM_2.5_ in 26 European cities, using the AirQ software from the WHO [10]. Davidson et al. [3] used Environmental Benefits Mapping and Analysis Program (BenMAP) from the US Environmental Protection Agency [11] to assess impact of PM_2.5_ reductions in the US. Boldo et al. [5] customized BenMAP to their Spanish setting to assess impact of PM_2.5_ reductions in all of Spain. Both Externalities of Energy model (ExternE) [8] and Evaluation of Air pollution model (EVA) [9] have used task specific models to make health impact assessments of the air pollution from European energy production and Danish emitted air pollution, respectively. These two HIA-tools, which in concept are most similar to the HIA model used in this study, are based on an impact pathway approach, where the impact of air pollution is assessed by a series of connected calculations, from amount and distribution of air pollution emitted by sources, via dispersion and chemical conversion, to air pollution exposure and health response experienced by the population, and to valuation of the health impact.

In this paper, we describe and quantify the impact of air pollution from Danish sources on human health in Denmark by means of an HIA modeling, using a newly developed HIA-model described by Flachs et al. [12]. The model was used as a Health Impact Assessment tool in the Centre for Energy, Environment and Health (CEEH) [12, 13], where a series of connected models covered the impact pathway of air pollution from the emitting sources, atmospheric dispersion, chemical transformation, and deposition, and the resulting impact on the exposed human population have been built.

The impact of air pollution scenario presented here is a modeling of the impact on the health in Denmark from all Danish sources from 2005 to 2030. Health outcomes included were incidence and mortality from lung cancer, coronary heart disease, stroke, and chronic obstructive pulmonary disease (COPD), as well as total mortality of other causes, as these had well-documented relations to air pollution [2] and formed a substantial amount of the morbidity and mortality in Denmark [14].

The Danish population is characterised by low child mortality and a life expectancy of 77.3 years for men and 81.6 years for women in 2011. In addition, it is characterized by large WWII birth cohorts in the 1940'ties and consequently large birth cohorts around a generation later in the 1960'ties. During the last decades, the birth cohorts have declined with a low point in 1982. Combined with increase in survival among those aged 65+ years, the population underwent and is expected to undergo further changes in age distribution (from 14.8% in 2000 to 24.4% above 64 years predicted in 2050). Combined with disease incidences that increase with age (e.g., coronary heart disease from 57 per 100,000 at the age of 30 to 2,937 per 100,000 at the age of 80 (average rates in 2000–2006)), the changes in age distribution alone indicated an increasing disease burden in the foreseeable future.

As air pollution is known to increase the risk of various diseases and mortality [2, 15–17], the underlying pattern of disease incidence, mortality, and demography played a key role in determining the resulting health impact of air pollution. In addition, most diseases do not have immediate onset but have clinical onset even years after exposure to risk factors, which meant that we had to take future developments in disease incidence, mortality, and demography into account, when assessing the health impact of air pollution.

This study aimed at quantifing and describing major health impacts of Danish emitted air pollution from 2005 to 2030 on the Danish population in the period 2005–2050 and to explore how the ageing of the Danish population in the same period influenced this health impact.

## 2. Materials and Methods

The HIA modeling was the final step in the CEEH-model chain, which started by modeling air pollution emissions from a set of assumptions on (worldwide) economic and technological development and development in the fuel and CO_2_ prices. This modeling was performed with the Balmorel model [18] developed at the Technical University of Denmark. The resulting air pollution emissions were then passed on to the Danish Eulerian Hemispheric model (DEHM model) [19] (Aarhus University Department of Environmental Science and Atmospheric Environment), an atmospheric chemical transport model. The DEHM model calculated the dispersion of air pollutants in the atmosphere, the chemical transformations, and the deposition. Calculations were carried out in a 16.6∗16.6 km grid over Denmark at an hourly basis. This allowed for calculations of yearly mean values of air pollutants over Denmark. These were recalculated via a transformation scheme as average yearly means for the 99 municipalities in Denmark rather than by grid. These municipal yearly means were used as the air pollution exposure for the population living in the municipalities. The HIA model then assessed health impacts and health economic consequences of the calculated air pollution exposure. This was done by linking air pollution exposure to changes in disease incidence by relative risks described by Bønløkke [2]. The model included four disease groups (lung cancer, coronary heart disease, stroke, and COPD) and mortality from all other causes.

In this study we examined the effect of an ageing population by comparing health impact assessments of the following three different population development scenarios.

 Fixed population kept incidence and mortality at the initial 2005 level and fixed the age and sex structure of the population at the 2005 level as well.
 Static mortality is a scenario where disease incidences and mortality remained unchanged at the initial 2005 level, but the population was followed as a cohort.
 Expected development is a scenario, which expanded the static mortality scenario by including a projected decline in disease incidences and mortality.

The air pollution exposure during 2005–2030, initial 2005 population, birth, and migration rates and all other parameters were kept equal between the three assessments. The difference between the first and second scenario was that, while the population was fixed in the *fixed population*, the *static mortality* allowed ageing of the birth cohorts and thus included potential effects of the large WWII birth cohorts and their children as described earlier. The *expected* scenario represented our expectation on the development of the Danish population, as defined by our projection of mortality and migration from 2005 to 2050. Note that the *fixed population* scenario was of no predictive value but was included to assess the impact of disparity in birth cohort sizes, as observed in the Danish population.

## 3. Model Description

The HIA model used was a multi state Markov model with states describing the health status for each one-year age and sex group. It consisted of a healthy state, first disease year state, a series of disease states after first year, and cause of specific death states for each of the four disease groups included, with a state to handle death from all other causes. For each state transition, probabilities to other states were established using Danish register data (see Section 4.2). Using coronary heart disease (CHD) as an example, we had the following states: healthy, disease in first year, disease in second year,…, disease in tenth year, dead from disease in first year, dead from disease in other years. The model included a set for each of the four diseases: coronary heart disease, lung cancer (limited to 8 disease years, due to high mortality), stroke, and COPD, and an additional state: dead from other causes. The model then considered each of the 99 municipalities in Denmark independently, to account for local variations in air pollution exposure, disease incidence, and mortality.

When evaluating the health impact of air pollution from 2005 to 2030, the modeled period was extended to 2050 allowing for time lags between exposure and disease onset. Each of the three scenarios were assessed by comparing two model runs: first a model run using the projected transition probabilities, and then a second model run, where the transition probabilities were modified by relative risks according to the impact of the modeled air pollution.

The full model methodology was described in detail by Flachs et al. [12].

## 4. Data

The HIA model required a number of different data to establish initial population, migration rates between model units (municipalities), morbidity and mortality rates, measures for associations between air pollution exposure and morbidity and mortality, and air pollution levels as population exposure.

### 4.1. Population Data

The model used initial (year 2005) one-year age and sex stratified Danish population at the municipal level. These were readily available from Statistics Denmark [20]. In order to account for intermunicipal migration which differed considerably between age groups (young adults primarily migrated towards larger cities and middle-aged adults migrated in the reverse direction), we used data on inter-municipal migrations also from Statistics Denmark. We used observed numbers for the last five year's net migration (2006–2011), a linear development in numbers towards Statistics Denmark 2030 projection, and finally that level from 2031 onwards. Observed (for 2005–2011) and expected numbers of births (2012–2050) at municipal level were also available from Statistics Denmark.

### 4.2. Incidence and Mortality

The model included four major disease groups: lung cancer (ICD-10 codes: C33-34), CHD (I20-25), stroke (I60-69), and COPD (J41-44) in addition to total mortality from other causes.

Data on incidence and cause of specific mortality were derived from the National Patient Register [21], the Danish Register of Causes of Death [22], and the Civil Registration System [23]. By linking the entire Danish population (aged 16 or more) each year from 1978 to 2006 between the three registers, we could year by year identify cases of the four relevant disease groups. Incidence probabilities for each year could then be calculated from the yearly incident cases, with a washout period of 16 years, and the midyear disease-free population. We then used a five-year (2001–2006) average as incidence rate for 2005 in the HIA model. By following the incident cases in the registers, we could then calculate yearly death probabilities among cases for each year following the incidence year for 10 years (8 years for lung cancer, as mortality is very high). All incidence and disease mortalities were calculated in sex-specific 10-year age groups, beginning at the age of 30. Overall, mortality and other causes of mortality probabilities were derived from the Danish Register of Causes of Death and calculated in sex-specific 1-year age groups.

The expected decrease in mortality in the *expected development* scenario was estimated from past mortality data from The Danish Causes of Death Register, as the mean decrease in a log-linear model of overall mortality from 1990 to 2005. The decrease was estimated for each sex in five-year age groups (except newborns and 1-2 year old each with their own level), and the initial (1990) level of mortality was estimated in one-year age groups. To ensure an expected reduction in overall mortality in the modelled period, we used a minimum yearly reduction of 2% in our projections of mortality from 2005 to 2050, as we expect all age groups to contribute to the mortality decline and a minimum of 2% brings the life expectancy to 86 and 88 years for males and females, respectively, in 2050.

In order to reduce mortality from the four chosen disease groups in line with the mortality from other causes, we also reduced the disease incidences by the same yearly percentages as the overall mortality.

**Figure 1:**
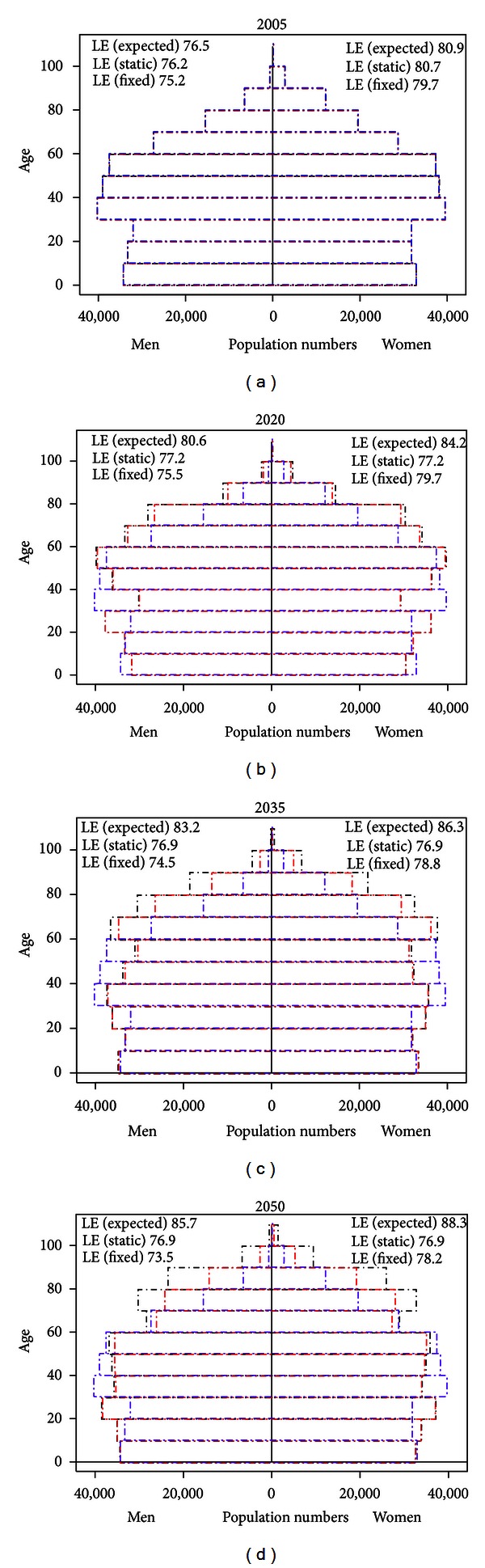
Expected demographic development of the Danish population 2005–2050 under the three scenarios. Black lines denote the *expected development* scenario, red lines the *static mortality* scenario, and blue lines the *fixed population* scenario.

The development of the age distribution and life expectancy of the population under the three different scenarios is presented in Figure 1. We note that for both men and women the *fixed population* and *static mortality* scenarios have fewer persons in the older age groups, and that the development is similar for both sexes, albeit more pronounced for women.

### 4.3. Air Pollution

Air pollution exposure was modeled by the DEHM model [19] as municipal averages of yearly mean ambient species (in *μ*g/m^3^). We focused on two main species: particulate matter (PM) with a diameter less than 2.5 *μ*m (PM_2.5_) divided into primary PM_2.5_ and secondary PM_2.5_ (nitrate and sulfate salts) and NO_2_, as these had well-documented impacts on human health and quantifiable associations (by relative risks) with incidences for specific disease groups. The underlying assumption on the association between air pollution and health through changes in disease incidence and mortality was that yearly average air pollution levels at peoples home address were an adequate measure of exposure. The studies, from which our relative risk estimates were drawn, were based on this assumption [2, 15–17].

**Figure 2:**
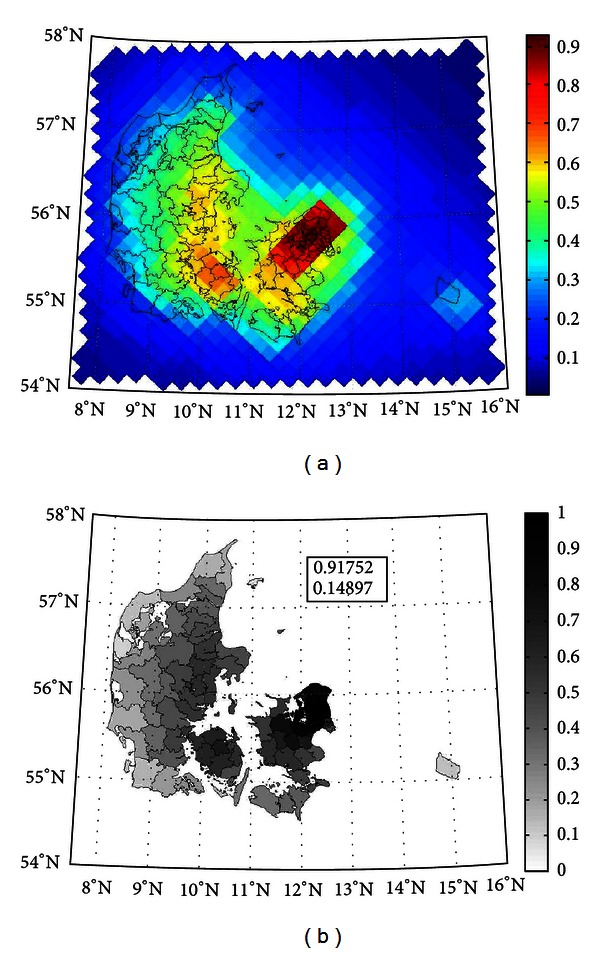
DEHM-modeled yearly average ambient PM_2.5_ from Danish sources at (a) grid and (b) municipal level in 2005. The scales in both plots are relative, and the absolute values for minimum and maximum (in *μ*g/m^3^) are in the box in the municipal plot.

We modeled the health impact of air pollution from Danish sources in the period from 2005 to 2030, sources included in the modeling were central power and heating plants, domestic decentralised heating, combustion in manufacturing industry, production processes, extraction and distribution of fossil fuels and geothermal energy, solvents and other product use, road transport, other mobile sources and machinery, waste handling and incineration, and agriculture. The population was exposed from 2005 to 2030, and the modeling of impact extended to 2050 because of important time lags between exposure and health impact. Simulation of air pollution exposure for a single year took about one week, and thus due to restrictions on available computational resources, we only had DEHM-model calculations for the specific years 2005, 2020, and 2030. We therefore linearly interpolated air pollution levels for years in between these. An example of the modeled air pollution is shown in Figure 2, which presents modeled yearly average mean ambient PM_2.5_ in 2005 and the transformation from DEHM grid to municipal level, as it may be seen that the transformation preserved the distribution of air pollution.

**Figure 3:**
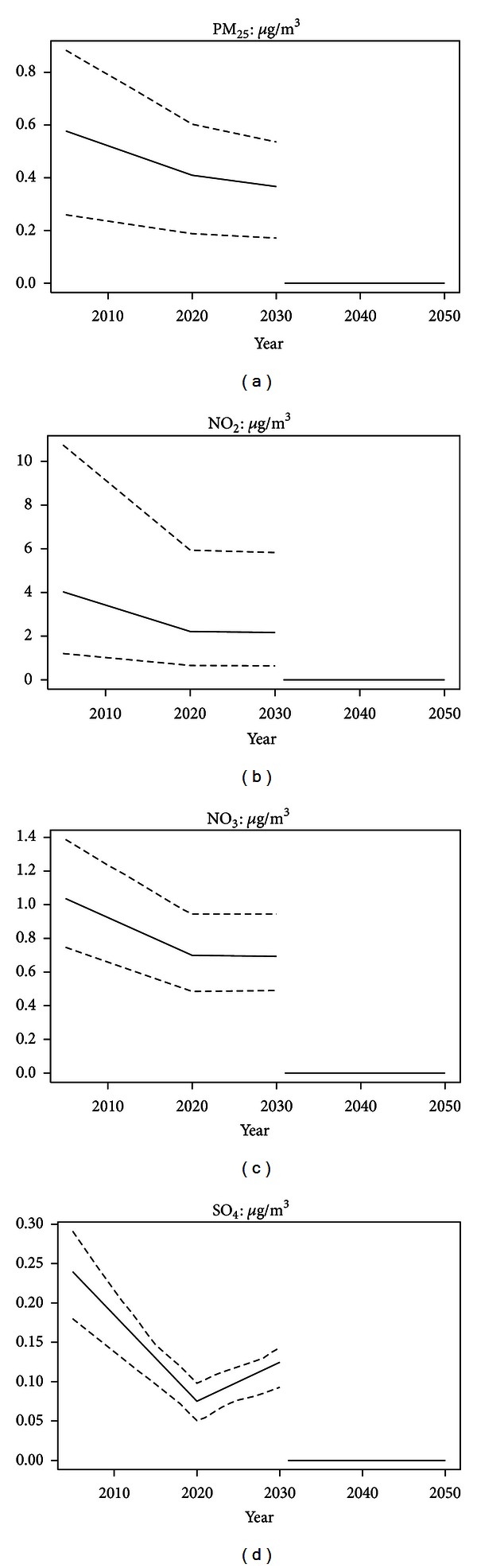
Development of Danish emitted air pollution exposure 2005–2030. Means, 5%, and 95% percentiles of the municipal levels in *μ*g/m^3^ for the four species. The zero level after 2030, shows that exposure after 2030 is not included in the modeling.

Figure 3 shows how exposure to the four chemical species developed from 2005 to 2030, presented as unweighed means, 5% and 95% percentiles of the municipal levels. This showed both the variation between municipal levels of exposure and the general tendency of decreasing air pollution levels from the 2005 level towards 2020, followed by an almost level development to 2030. These general trends, however, covered a wide disparity between developments in the municipalities and exposure to SO_4_ which tended to increase from 2020 to 2030, albeit not to the 2005 level of exposure.

### 4.4. Relative Risks

The relative risks used to associate exposure to air pollution with changes in disease incidence were discussed by Bønløkke [2]. We chose to follow Pope et al. [15] for lung cancer, Abbey et al. [16] for COPD, and Miller et al. [17] for CHD and stroke, as these comprehensive studies followed large cohorts for many years. Several studies supported that males may be at significantly lower risk than females [24, 25], and as the relative risks observed by Miller et al. [17] only applied to women we decided to reduce the relative risks for men by half with regard to the diseases studied by Miller et al. [17], even though we modeled incidence and not mortality.

In addition, we included a relative risk for death from other causes, as the four considered diseases did not account for the entire change in mortality associated with air pollution. It was based on the relative risk for mortality from all causes from Pope et al. [15] (RR = 1.006), and then halved to account for the causes of death modelled directly in the HIA model. If the full relative risk of 1.006 had been included, we would have double counted deaths, as we modeled mortality from four major disease groups directly. These groups accounted for around half of the total mortality, and consequently that factor was used. See Table 1 for actual values.

### 4.5. Lead and Lag Times

In order to model a realistic development of occurrence of disease and mortality, we introduced a set of lead and lag times, to delay onset of incidence and mortality from timing of exposure to air pollution. The lead time was the number of years from exposure to maximum elevated risk, and lag time was the number of years from maximum elevated risk to no excess risk due to air pollution exposure. Our choice of lead and lag times was based on the discussion of lead and lag times for smoking in Baan et al. [26]; exact figures are presented in Table 1.

### 4.6. Health Care and Labour Market Cost

 The health economic cost associated with changes in air pollution exposure and incidence of diseases was calculated as a sum of two parts: (1) a contribution from changes in health care use calculated as attributable cost of the specific diseases, and (2) a contribution from consequences from the labour market due to reduced wages, absence, or early retirement caused by changes in disease incidence and mortality calculated by human capital methodology. The approach, methodology, and actual calculations of unit prices for different states in the model were documented in detail in Sætterstrøm et al. [27] for health care costs and in Kruse et al. [28] for labour market consequences. Aggregations of social costs used a 3% discount rate.

## 5. Results

**Figure 4:**
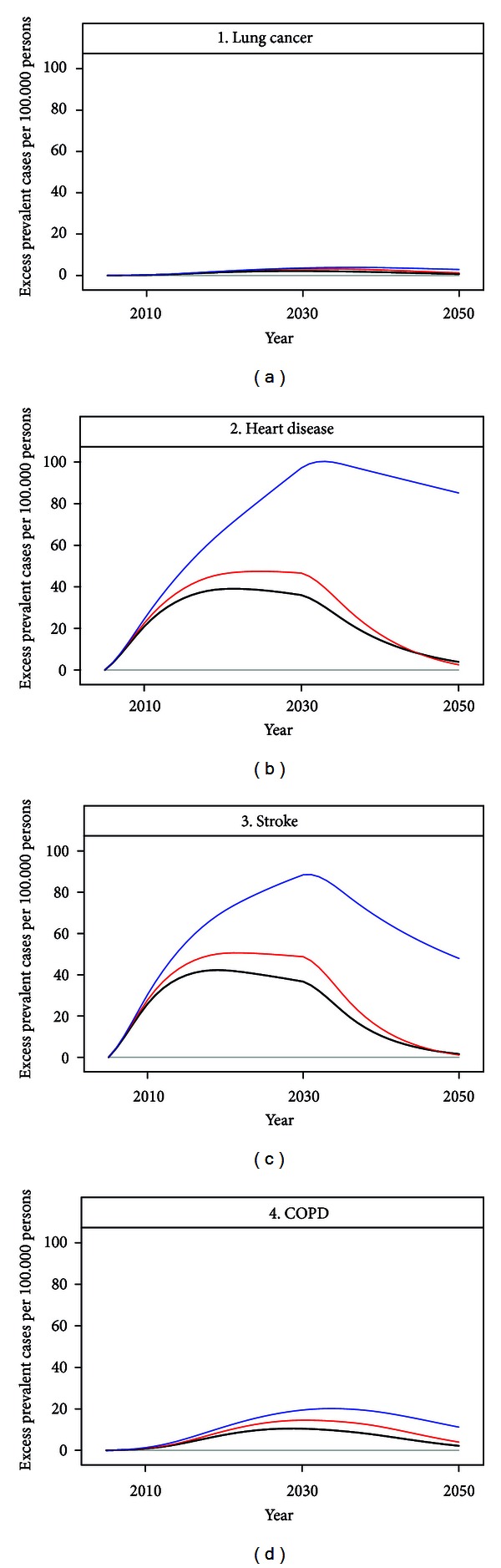
Summary of yearly excess prevalent cases of diseases in 2005–2050 related to air pollution from Danish sources emitted in the period 2005–2030, for the four selected disease groups (in numbers per 100.000 persons). Black lines denote the *expected development* scenario, red lines the *static mortality* scenario, and blue lines the *fixed population* scenario.

Figure 4 depicts the impact in Denmark of Danish emissions of air pollution on the number of prevalent disease cases in 2005–2050 under the three different scenarios. We found that the *fixed population* scenario had the most impact on the disease prevalence, and the *expected development* had the least impact for all diseases. The impact in all scenarios reflects both the incidences which were low for lung cancer and COPD and high for CHD and stroke, and the lethalities which were very high for lung cancer and much lower for the three other diseases. In addition, we found that even 20 years after the last pollution exposure, there was still an effect of former air pollution, as disease cases attributable to air pollution were still alive (heart disease and stroke).

**Figure 5:**
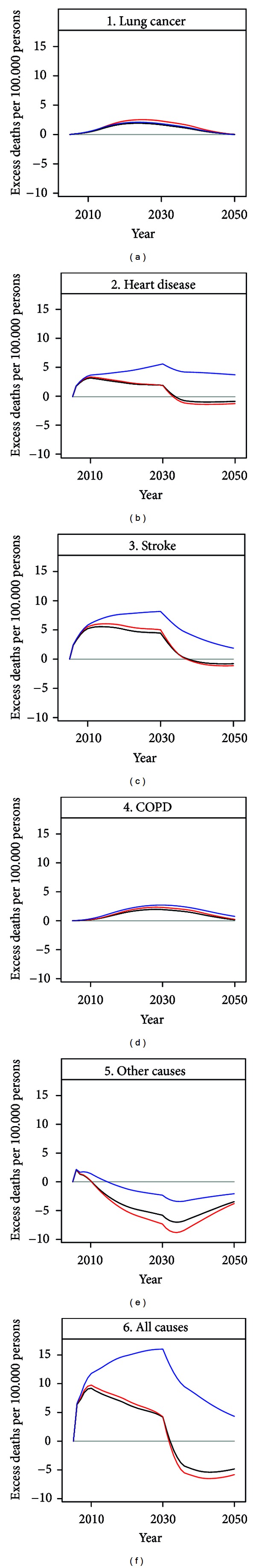
Summary of yearly excess deaths in 2005–2050 related to air pollution from Danish sources emitted in the period 2005–2030, for the four selected disease groups and other cause mortality (in numbers per 100.000 persons). Black lines denote the *expected development*, red lines the *static mortality* scenario, and blue lines the *fixed population* scenario.

Figure 5 shows the expected development in mortality under the three different scenarios. Not surprisingly the *static mortality* and *expected development* scenarios had fewer deaths attributable to air pollution from CHD, stroke, and COPD. Air pollution impact on lung cancer mortality was more or less similar between the three scenarios. The sharp decline in excess mortality just after 2030 was explained by the end of air pollution exposure in 2030. The two scenarios *expected development* and *static mortality* both have negative excess deaths for heart disease and stroke in the last part of the modeled period, which could be explained as the effect of the “harvesting” in the period with air pollution exposure, leaving fever individuals to die after a period with increased mortality. The *fixed population* scenario did not experience this “harvest” effect, as the population was the same each year.

The negative numbers of excess other cause mortality could be explained by the increase in cause specific mortality, as people only die once. The scenario *fixed mortality* had markedly less other cause mortality, again because the “harvest” effect was not in play here.

**Figure 6:**
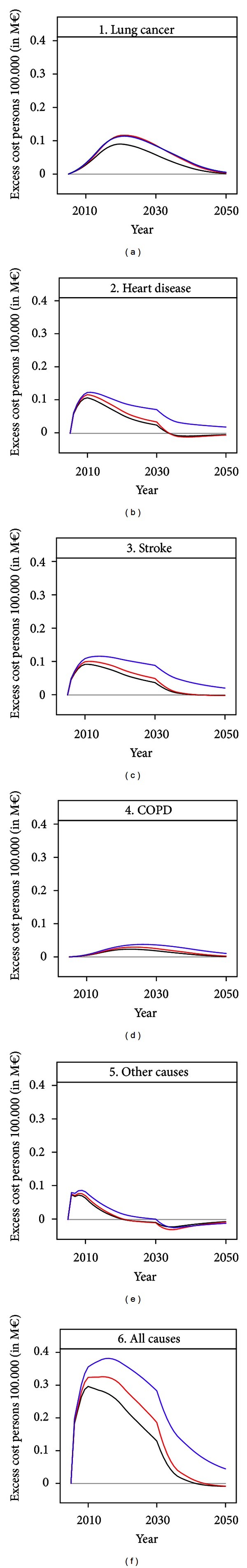
Summary of yearly health care and labour market costs in 2005–2050 related to air pollution from Danish sources emitted in the period 2005–2030, for the four selected disease groups and other causes of mortality (in m€ per 100,000 persons). Black lines denotes the *expected development* scenario, red lines the *static mortality* scenario, and blue lines the *fixed population* scenario.

Figure 6 depicts the development in health related costs for the three modelled scenarios. Compared to the mortality summary in Figure 5, we noted that costs for all disease groups and overall mortality were consistently lower under the *expected development* scenario, compared to the *static mortality* scenario.

In Table 2, yearly excess deaths, lost life years, and social costs are summed up for the entire modeled period from 2005 to 2050 for the three scenarios for population development using a 3% discount rate. Even though women lost more life years and have more premature deaths, they incurred lower costs, which could be explained by the lower unit cost for women leaving the labour market or dying, as women in general had lower wages than men and thus incurred less productive loss when not attached to the labour market [28].

The figures in Table 2 further underlines the marked differences between the results of the three scenarios, where the the *fixed population* scenario had a 65% higher cost (244.5 + 185.9 M€ versus 153.6 + 108.0 M€) and 491% more excess deaths (10.355 + 16.559 versus 2.134 + 2.416) than the *expected development* scenario, while the *static mortality* scenario had 21% higher cost (183.1 + 134.4 M€ versus 153.6 + 108.0 M€) and 7% fewer deaths (1.816 + 2.434 versus 2.134 + 2.416) compared to the *expected development* scenario.

As number of excess deaths tended to zero as time passed, it was highly dependent on timing of calculation. The number of lost life years is a measure of health impact which is less timing dependent, and as such a better measure of lost life. The Danish emitted air pollution in 2005–2030 resulted in 672,323, 290,273, and 280,258 lost life years in the three scenarios, respectively, with the largest losses incurred among women. Detailed information on sex and age distribution is available in Table 2.

## 6. Discussion

 The HIA model used here to quantify the impact of Danish emitted air pollution presented a novel way of making health impact assessment of air pollution, by using a bottom-up setup, where the exposed population was followed up, and current health status was modeled. The population dynamics and developments in disease incidence and mortality were thus directly incorporated into the modeling, in contrast to traditional health impact assessment which more or less explicitly used a top-down setup, with a fixed population and no population dynamics [3, 4, 7–9]. In addition, we introduced the possibility of modeling the health impact of a series of years with differing exposure. Life-table assessments also allowed for calculation of several years of exposure and for changing levels of exposure; however, they did not, to our knowledge include population dynamics caused by other changes in demography than those associated with air pollution [29], and most other models either just assessed the impact of one year at a time [8, 9] or made an assumption of a stable level of air pollution exposure [3, 4, 7].

The four included disease groups were chosen because of their well-documented relation to air pollution and because they are important diseases and causes of death, thus with a profound effect on population health and mortality. In 2005, coronary heart disease, lung cancer, stroke, and COPD together comprised around half of all causes of death in Denmark [22]. The differences in relative risks for men and women used here were based on studies of mortality and emissions of particulate matter [15–17, 24], with an assumption of a halved risk for men compared to women [24, 25]. The rather large relative risks, particularly in Miller et al. [17], are in accordance with leading groups of experts in the field of modeling of effects of air pollution [30].

Our assumption on the relative risk of other causes of mortality (1.003 per *μ*g/m^3^ increase) as half the risk of all-cause mortality (1.006 per *μ*g/m^3^ increase) for PM_2.5_, NO_2_ and SO_4_ was included to account for the fact that even after modeling the four major disease groups and their associated mortality, the model would not account for all the expected increase in total mortality, thus some remnant excess risk of death persists. In order to account for this, we included an albeit rough estimate of half the relative risk from death of all causes. The exact size of this remnant effect is open to discussion, but entirely omitting the effect will lead to a serious underestimation of the health effects of air pollution. Evidence and expert judgements are suggestive of an even larger total effect than a change in relative risk of 1.006 [1, 31], possibly almost twice as high. One of the lines of evidence for a larger effect is the fact that studies with more precise exposure estimates tend to show stronger effects such as the American Cancer Society Study and the Seventh-Day Adventist Health Study on smog from which the original lung cancer and chronic bronchitis effects stem [15, 16]. The study by Abbey et al. [32] rely on exposure data that are general for large geographical areas and imprecise on the individual level. Good estimates from a street level model in Denmark with historical air pollution data at address level have provided data on changes in relative risks associated with NO_2_ for lung cancer and COPD, and a strong effect of air pollution on COPD was recently observed in Denmark [33, 34]. We therefore decided to apply these estimates in addition to the commonly used conservative PM estimates rather than increasing the responses of the latter as suggested by the expert judgments.

The lead and lag times used in the modeling were derived from a comprehensive study on the relation between exposure to smoking and disease and mortality in Baan et al. [26]. As no studies were available on air pollution and lead and lag times, we used these, as the exposure was similar, albeit of much larger extention in smoking, which leads to the same diseases. This approach was also used in Leksell and Rabl [35]. The shapes of the curves in Figure 5 were to a large extention determined by our chosen lead and lag times, but as the total excess risk in the model was determined by amount of exposure to air pollution and unaffected by lag and lead times, the total impact was unchanged, only the timing of events changed with changes in lead and lag times.

The negative numbers of excess deaths in the oldest age group in Table 2 and in the last period of the modeling (2040–2050) might be explained by people exposed to air pollution dying prematurely (and younger) in the beginning of the modeling period, and thus missing as old persons in the last part of the period, that is, the harvest effect as described by Rabl [36].

The effect of the human capital method was seen clearly in Table 2, where the age groups above 64 years contributed relatively less to the social cost compared to the age groups 0–39 and 40–64 years. This was even more pronounced when the number of excess deaths by age groups (see Table 2) was taken into account. Death and disease among persons above 64 years did not incur any productivity loss as the Danish retirement age of 65 was used in the calculations of unit costs, and thus the only cost included for death and disease among people above 64 years was the attributable health cost, which was minor compared to the cost of production loss among younger people.

When our modeling was contrasted against another modeling of air pollution [4, 6, 7, 9], it was essential to remember that the other modeling had both differing air pollution and/or differing populations, and thus the actual health impacts and associated costs were difficult to compare. Even within our modeling, where the initial population and the air pollution exposure were equal across scenarios, changes in mortality development lead to either a 65% or a 21% increase in cost depending on demographic dynamics compared to the *expected development* scenario.

When comparing our social cost of 261.6 M€ from the *expected development* scenario to the social costs of 817 M€ (2000 figure) by Brandt et al. [9], which was the most comparable assessment, as it was done on Danish emissions and the Danish population, we found a significantly lower amount. This was primarily due to three factors. Firstly, Brandt et al. used a different cost model to calculate unit costs for diseases and mortality. In both HIA models, costs were a sum of health care costs and a cost of lost life, the latter calculated as either a production related labour market consequences component or a willingness to pay for lost life component. The lost life component was by far the dominating cost component, and as people after retirement (at age 65) do not incur any production losses when falling ill or dying, there was only the health care component left in our model, whereas Brandt et al. [9] calculated cost of lost life by a willingness to pay method, leading to generally higher costs, and particularly for the postretirement age group, which incurred a cost by this method. Secondly, Brandt et al. [9] assessed the health impact of a single year of air pollution exposure with an underlying assumption of no population dynamics, whereas our *expected development* took births, mortality, migrations, and municipal differences in these into account in an integrated dynamic modeling. It was worth nothing that the *fixed population* scenario, which was the closest scenario to the one in Brandt et al. [9], yields a cost of 430.4 M€, which was much closer to the 817 M€ figure in Brandt et al. Thirdly, the air pollution levels were on average higher in year 2000 which was modeled in Brandt et al. compared to the years following year 2005 in our model.

The ExternE [8, 37] also used an impact pathway approach to model single year air pollution impacts. ExternE modeled the entire European Union, though at a much less detailed level, and thus, for example, mortality rates were assumed equal across Europe. They reached a cost of 1,776 M€ for energy production related air pollution in Denmark alone for 1995 [38], which was much higher than our 261.6 M€. The ExternE figure includes a price on CO_2_ emissions, which might explain some of the difference, but the main difference in methodology must again be ascribed to their use of willingness-to-pay methods to value lost life. It is worth noting, that both Brandt et al. [9] and ExternE [8] included many more diseases in their modelings but concluded, like we did, that the cost was mainly driven by the cost of lost life, and those are included in both our and their model.

The three different population scenarios gave widely differing results, with especially the *fixed population* scenario having a much larger impact independently of health impact measure. This was expected, as the *static mortality* and the *expected development* scenarios were much more alike in the demographic dynamics, as they both followed the year 2005 Danish population from 2005 to 2050, with only disease incidences and mortality setting them apart. The *fixed population* scenario, on the other hand, each year had the same year 2005 population, and only changes in air pollution exposure. This leads to a pronounced lack of harvest effect and to an inflation in the number of persons in disease states with low mortality, as is clear from Figure 4, where lung cancer and COPD were much less inflated compared to heart disease and stroke. The scenario was not realistic in any way but was the closest one we could get to follow a population without introducing any ageing effects at all and was included for comparative reasons. In contrast to what was expected, the scenario with the least impact in terms of overall deaths was the *static mortality*, in spite of the *expected development* scenario having far more elderly people and thus more people in the high risk ages. This effect might be explained by the much stronger harvest effect in the *static mortality *scenario, and when looking at lost life years we saw that the *expected development* scenario yielded fewer lost life years than the two other scenarios. The explanation was that while the number of elderly persons was the highest in the *expected development* scenario their general health status, expressed by the incidence and mortality risks, was in fact better due to the expected general improvements in public health. Thus when modeling impacts from exposures with long-term effects, population dynamics played a key role.

It was worth noting that the impact of Danish emitted air pollution (1011 premature deaths in 2010, where the impact on mortality was the largest in the *expected development* scenario) was of a small magnitude compared to other risk factors such as smoking and alcohol, which, respectively, accounted for around 15,000 and 3,000 premature deaths annually (2005 figures) [14]. Our modeling only considered Danish emitted air pollution, and the total health impact of air pollution would be four to five times higher, as Danish emitted air pollution is around 20%–25% of the total air pollution in Denmark.

## 7. Conclusion

Assumptions on population development leading to different ageing patterns played a key role in assessing the health impact of air pollution, and the population dynamic effects were important when making assessments of health impacts that occur with a considerable delay, as demonstrated with the different costs of Danish emitted air pollution in the period 2005–2030 of 430.4, 317.5, or 261.6 M€ depending on inclusion of population dynamics in the health impact assessment.

## Figures and Tables

**Table 1:** ICD-10 codes for disease groups, lead and lag times, and relative risks for association between air pollution and incidence and mortality. All relative risks are per **μ**g/m^3^ increase in yearly average ambient species concentration.

Chemical species disease	ICD-10 codes	Lead time	Lag time	Relative risk	
				Male	Female
PM_2.5_, NO_3_, SO_4_					
Coronary heart disease	I20–25	1	5	1.0105	1.021
Stroke and cerebrovascular disease mortality	I60–69	1	5	1.0175	1.035
Lung cancer	C33-34	10	15	1.014	1.014
COPD	J41–44	10	15	1.014	1.014
Other cause mortality		0	0	1.003	1.003
NO_2_					
Coronary heart disease	I20–25	1	5	1.0^*∗*^	1.0^*∗*^
Stroke and cerebrovascular disease mortality	I60–69	1	5	1.0^*∗*^	1.0^*∗*^
Lung cancer	C33-34	10	15	1.008	1.008
COPD	J41–44	10	15	1.005	1.005
Other causes of mortality		0	0	1.0^*∗*^	1.0^*∗*^

Sources: lead and lag times: Baan et al. [26]. Relative risks: Abbey et al. [16], Pope et al. [15], Chen et al. [25], Miller et al. [17], and Bønløkke [2].

*: no effect included in the model.

**Table 2:** Total numbers of excess deaths, lost life years, and social cost in M€ for the years 2005–2050 attributable to Danish air pollution emitted in the period 2005–2030 by age and sex, for the three different scenarios (first: *fixed population*, second: *static mortality*, and third: *expected development*).

		Men			Women	
	Fixed	Static	Expected	Fixed	Static	Expected
Excess deaths						

0–39 years	165	116	79	133	77	51
40–64 years	2.808	1.824	1.431	2.838	1.778	1.278
65–84 years	5.995	3.655	4.184	9.117	5.925	5.762
85+ years	1.387	−3.779	−3.560	4.471	−5.346	−4.676

Total	10.355	1.816	2.134	16.559	2.434	2.416

Lost life years						

0–39 years	4.670	1.546	1.102	3.498	977	698
40–64 years	65.903	14.921	11.618	64.044	13.747	9.898
65–84 years	146.736	70.976	62.391	224.341	80.010	66.318
85+ years	38.446	36.256	46.562	124.683	71.839	81.672

Total	255.756	123.699	121.672	416.567	166.574	158.586

Social cost (M€)						

0–39 years	10.6	8.1	6.0	7.0	4.8	3.5
40–64 years	197.0	144.9	120.2	118.3	87.4	67.4
65–84 years	33.5	37.0	33.3	50.5	48.8	41.9
85+ years	3.5	−7.0	−5.9	10.1	−6.6	−4.9

Total	244.5	183.1	153.6	185.9	134.4	108.0
